# Disease Stage-Associated Alterations in Learning and Memory through the Electroacupuncture Modulation of the Cortical Microglial M1/M2 Polarization in Mice with Alzheimer's Disease

**DOI:** 10.1155/2020/8836173

**Published:** 2020-08-28

**Authors:** Long Li, Le Li, Jiayong Zhang, Sheng Huang, Weilin Liu, Zhifu Wang, Shengxiang Liang, Jing Tao, Lidian Chen

**Affiliations:** ^1^College of Rehabilitation Medicine, Fujian University of Traditional Chinese Medicine, Fuzhou, Fujian 350122, China; ^2^Fujian Key Laboratory of Rehabilitation Technology, Fuzhou, Fujian 350122, China; ^3^National-Local Joint Engineering Research Center of Rehabilitation Medicine Technology, Fujian University of Traditional Chinese Medicine, Fuzhou, Fujian 350122, China; ^4^College of Integrated Traditional Chinese and Western Medicine, Fujian University of Traditional Chinese Medicine, Fuzhou, Fujian 350122, China

## Abstract

Microglia are the primary cells that exert immune function in the central nervous system, and accumulating evidence suggests that microglia act as critical players in the initiation of neurodegenerative disorders, such as Alzheimer's disease (AD). Microglia seemingly demonstrate two contradictory phenotypes in response to different microenvironmental cues, the M1 phenotype and the M2 phenotype, which are detrimental and beneficial to pathogenesis, respectively. Inhibiting the M1 phenotype with simultaneous promoting the M2 phenotype has been suggested as a potential therapeutic approach for cure AD. In this study, we demonstrated that electroacupuncture at the Shenting and Baihui acupoints for 16 weeks could improve learning and memory in the Morris water maze test and reduce amyloid *β*-protein in the parietal association cortex and entorhinal cortex in mice with mild and moderate AD. Besides, electroacupuncture at the Shenting and Baihui acupoints not only suppressed M1 marker (iNOS/IL-1*β*) expression but also increased the M2 marker (CD206/Arg1) expression in those regions. We propose that electroacupuncture at the Shenting and Baihui acupoints could regulate microglial polarization and decrease A*β* plaques to improve learning and memory in mild AD mice.

## 1. Introduction

Alzheimer's disease (AD) is an age-related neurodegenerative disease that has become the fourth leading cause of death worldwide [1]. More than 50 million people are affected by dementia, and the total estimated cost of dementia is 818 billion dollars [2]. With the increasing aging population, AD has become an urgent problem to be solved for all humankind.

Over the past two decades, there has been a global cumulative investment of 600 hundred billion dollars to find a cure for AD, and more than 95% of drugs have failed in clinical trials [3]. A progressive cognitive decline clinically characterizes AD, but pathological changes in the brain precede clinical symptoms for several years. Given the previous failures, the Food and Drug Administration drafted a guideline for the development of drugs for early-stage AD [4, 5], and current paradigms of drug design must shift from a single-target approach to developing drugs targeted at multiple disease aspects. Acupuncture is a potential therapeutic procedure that has shown evidence for clinical efficacy and safety in the treatment of chronic pain [6] and a variety of symptoms and conditions associated with cancer [7]. Electroacupuncture (EA) has been widely used to treat neurodegenerative diseases, including dementia [8, 9]. EA could improve the Mini-Mental State Examination scores in patients with AD [10] and ameliorate cognitive deficits in animal models of AD [11, 12]. However, how EA improves cognitive function is still unknown.

Neuroinflammation has an essential impact on neurodegenerative diseases. EA is a nerve stimulation therapy that uses immunomodulation and controls inflammation to reestablish physiological homeostasis during illness. A previous study found that EA might play a neuroprotective role [13] by enhancing anti-inflammatory activity to suppress aberrant glial activation and prevent the loss of neurons in neurodegenerative disorders [14]. Microglia, the resident immune cells of the mammalian CNS, play a pivotal role in neuroinflammation. In a healthy brain, microglia are involved in modulating higher cognitive functions such as learning and memory [15, 16]. However, the function of microglia may be dynamic because of the different pathological stages of AD. Microglia are classified as ramified (quiescent/resting), activated, or ameboid (phagocytotic). There is a model that describes the mechanism of two opposite polarization states of macrophage activation: the classically activated proinflammatory M1 macrophages and the alternatively activated anti-inflammatory M2 macrophages. The macrophage nomenclature was adopted to describe the different functional states of microglia [17]. Microglia have complex roles that are detrimental and beneficial to AD pathogenesis: M1 phenotypes are characterized by the production of inducible nitric oxide synthase (iNOS) and inflammatory cytokines (such as IL-1*β*) and damage healthy cells, such as neurons, leading to A*β* accumulation; M2 phenotypes express mannose receptor (CD206) and arginase 1 (Arg1) and downregulate neuroinflammation and remove A*β* plaques.

In early-stage AD, A*β* oligomers, the hydrolysis product of amyloid precursor protein by *β*- and *γ*-proteinase, lead to the M2 phenotype activation and introduce cell factors to prevent the overproduction of A*β* and further pathological damage. However, the chronic A*β* deposition stimulates microglia persistently [18] and causes the M1 phenotype activation to increase. In 18-month-old mice, the microglial activation is expanded into hippocampal areas free of plaques, showing that classic M1 phenotypes produce cytotoxic effects on neurons [19]. The ablation of iNOS in APP/PS1 mice can protect mice from the plaque formation and premature mortality [20]. These contradictory functions of microglia reflect their acquisition of distinct M1/M2 phenotypes in response to different microenvironmental cues [21, 22]. A study found that naringenin promotes the microglial M2 polarization and A*β* degradation enzyme expression in AD [23], and the suppressor of cytokine signaling 3 suppresses the microglial polarization to the M1 phenotype by blocking the IL-6 production in vitro [24]. Therefore, with microglia, as essential members of the CNS, the conversion of phenotype and functions may lead to a novel therapeutic strategy to cure AD.

Our previous studies have demonstrated that learning and memory impairment in AD model mice could be improved by EA [25, 26] EA could repress the activation of microglia in various pathological models [27, 28]. Recently, research showed that the transformation of microglia into an engulfing state could reduce A*β*, but overactivated microglia would accelerate the A*β* deposition in AD [29]. Therefore, we wondered whether EA could regulate the microglial polarization in different pathological stages. In the present study, we chose 4- and 12-month-old APP/PS1 mice to simulate mild AD and moderate AD, respectively, aiming to investigate whether EA could regulate the microglial polarization to modulate learning and memory at different stages of Alzheimer's disease.

## 2. Methods and Materials

### 2.1. Animals

Male APPSwe/PS1B6C3-Tg [*B6C3-Tg* (*APPswe*, *PSEN1dE9*) *85Dbo/MmJNju*] mice and female wild-type mice (C57/BL6) were purchased from the Model Animal Research Center of Nanjing University (SYXK2013-009) for breeding. Animals were housed 3-4 per cage in temperature (21–25°C) and light (12-h light/dark cycle) controlled rooms, with free access to food and water until the end of the experiment. All studies performed on mice were performed following the guidelines of the Fujian University of Traditional Chinese Medicine Animal Care and Use Committee, which approved all protocols used in this study.

### 2.2. Experimental Design

Data presented in this study were obtained in animals aged 4 months (mild AD) and 12 months (moderate AD). For mild AD mice, the intervention began at 4 months old and ended at 8 months old. For moderate AD mice, the intervention began at 12-month-old and ended at 16-month-old. The treatment lasted for 16 weeks. The Morris water maze (MWM) test was carried out twice before and after the intervention immediately. When trials were carried out, mice were sacrificed.

A total of 48 male APP/PS1 transgenic mice and 16 male wild-type mice were used in this study. According to age, mice were divided into mild AD and moderate AD. APP/PS1 mice were identified by polymerase chain reaction (PCR). According to the results of PCR, transgenic APP/PS1 mice were randomly divided into the AD group, the EA group, and the nonacupoint (NA) group. Wild-type (WT) littermate mice matched for age represented the WT group. There were 8 mice in each group.

### 2.3. Morris Water Maze Test

The MWM is a classical learning and memory behaviour test for rodents. The MWM device is a circular pool that is divided into four quadrants by a computer identification system to better record the performance of animals. In the place navigation test, mice were put into water to find a hidden platform within 90 seconds, which is located in the centre of the target quadrant. The mice were trained four times every day on four consecutive days. The platform was fixed for each mouse, but the swim-start position was randomized every day and alternated clockwise. The time each mouse took to find the platform within 90 seconds was recorded (escape latency). If the platform was not found within 90 seconds, the investigator guided it to the platform and kept it on the platform for 15 seconds, and the escape latency was recorded as 90 seconds. After the place navigation test, the space exploration test was conducted on the fifth day. The platform was removed, and the mouse was placed into the water from the contralateral quadrant. The number of crossings over the original platform within 90 seconds was counted to assess the learning and memory of mice.

### 2.4. Electroacupuncture Stimulation

After the baseline measurement of the MWM test, electrical stimulation was performed. Mice in the EA group received electrical stimulation at the Shenting (DU24) and Baihui (DU20) acupuncture according to our previous study [30]. DU24 was connected with the positive pole, and DU20 was connected with the negative pole. Mice in the NA group received electrical stimulation at both side nonacupoints (the area below costal region, 2 cm superior to the posterior superior iliac spine, and ~3 cm lateral to the spine). The left side was connected with the positive pole, and the right side is connected with the negative pole. Acupuncture needles (*φ*0.3 × 13 mm, Hwato) were connected to the EA apparatus (model G6805; Suzhou Medical Appliance Factory, Shanghai, China), with a disperse-dense wave. The frequency was 1/20 Hz, and the intensity of current was 1 mA. Mice were stimulated for 16 weeks, once every other day, 3 times a week, and the treatment lasted for 30 min each time. Mice in the AD group and the WT group were fed and grasped under the same conditions without any treatment.

### 2.5. Immunofluorescence

Mice were sacrificed after anesthesia via 4% paraformaldehyde perfusion. The brain was cut into 5 *μ*m thick slices after paraffin embedding. After dewaxing and tissue antigen repair, sections were incubated in 5% goat serum for 1 hour at room temperature and in primary antibody overnight at 4°C. The next day, sections were incubated with fluorescein secondary antibody for 2 hours. The primary antibodies and concentrations used in this experiment included anti-Iba1 (1 : 500; ab5076, Abcam), anti-iNOS (1 : 300, ab49999, Abcam), anti-IL-1*β* (1 : 100, ab9722, Abcam), anti-Arg1 (1 : 100, 16001-1-AP, Proteintech), anti-CD206 (1 : 200, ab8918, Abcam), and anti-6E10 (1 : 1000, 803001, Biolegend). The secondary antibodies used were as follows: donkey anti-goat-Alexa 594 (1 : 1000; ab150132, Abcam), donkey anti-mouse-Alexa 488 (1 : 1000; ab150105, Abcam), donkey anti-rabbit-Alexa 488 (1 : 1000; ab150061, Abcam), and donkey anti-mouse-Alexa 594 (1 : 1000; ab150108, Abcam). After referencing *The Mouse Brain in Stereotaxic Coordinates* [31], we chose the parietal association cortex (PtA, coordinates: -1.82 mm to -2.06 mm from bregma, coronal serial sections, taking every other section for analysis) and entorhinal cortex (Ent, coordinates: -2.92 mm to -4.24 mm from bregma, coronal serial sections, taking one out of every five sections for analysis) as the regions of interest (ROIs). Four views of each piece were randomly selected for capture. An inverted laser scanning confocal microscope (LSM780, Zeiss) was used for immunofluorescence colabeling imaging, and the numbers of positive cells were manually counted. A fluorescence microscope (DM4000 B, Leica) was used for A*β* plaque imaging, and the number of plaques and plaque area fractions in the observation field were calculated by the ImageJ software. All pictures and quantifications were performed by investigators who were blinded to the experimental grouping.

### 2.6. Quantitative Real-Time Polymerase Chain Reaction

Mice were decapitated after anesthesia, and the brain tissue was separated. In detail, total RNA was isolated from tissues with RNAiso plus and reverse transcribed into cDNA using the HiScriptIIQRT SuperMix reagent kit (R223-01, Vazyme). Finally, the cDNA was quantified by qPCR using a SYBR qPCR Master Mix (Q311-02, Vazyme). The mRNA expression levels were calculated by the 2^-*ΔΔ*Ct^ method after the normalization to the expression of M1 markers (iNOS/IL-1*β*), M2 markers (CD206/Arg1), or GAPDH. All experiments were performed in triplicate and repeated at least three times. Four mice in each group were used for statistical analysis.

### 2.7. Statistical Analysis

The SPSS 21.0 software was used to perform the data analysis. The experimental data are expressed as the mean ± SEM. Mauchly's test of sphericity was used to assess the correlation of repeated-measures data. Repeated-measures analysis of variance was performed for the place navigation test. One-way analysis of variance was applied for other data when assumptions of normality and equal variance (*F* test) were met; otherwise, the equivalent nonparametric test will be used. A value of *P* < 0.05 was considered statistically significant.

## 3. Results

### 3.1. Electroacupuncture Improved Learning and Memory in APP/PS1 Transgenic Mice

**Figure 1:**
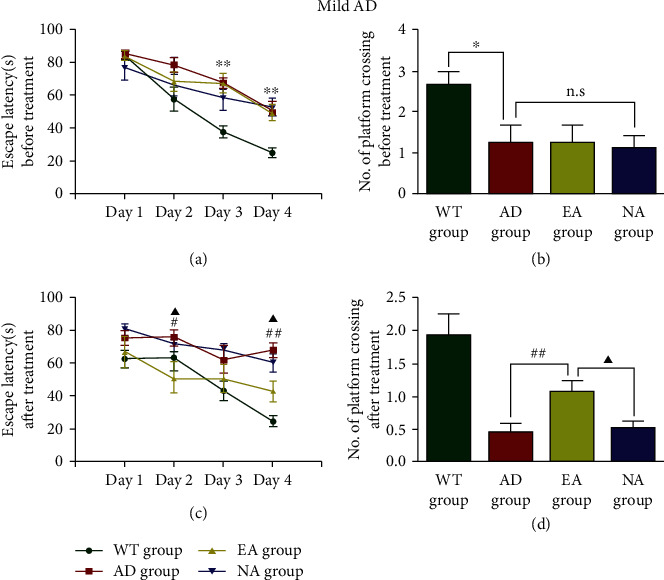
The Morris water maze test in mild AD mice. (a) Escape latency and (b) the number of platform crossings before treatment. Escape latency (c) and the number of platform crossings (d) after treatment. *N* = 8/group, WT group vs AD group, ^∗^*P* < 0.05, ^∗∗^*P* < 0.01. AD group vs EA group, #*P* < 0.05, ##*P* < 0.01. EA group vs NA group, ▲*P* < 0.05, n.s. means no significant difference.

First, we determined whether the EA stimulation could delay/reverse learning and memory impairment in APP/PS1 transgenic mice. In mild AD, the escape latency was decreased (*P* < 0.01, Figure 1(a)), and the number of platform crossings was increased (*P* < 0.05, Figure 1(b)) in the WT group compared with the AD group in the MWM test. There was no significant difference between the AD group, the EA group, and the NA group at baseline. After 16 weeks of intervention, the escape latency of the EA group was decreased comparing with the AD group (*P* < 0.01, Figure 1(c)), and there was a significant difference between the EA group and the NA group (*P* < 0.05). In the space exploration test, mice in the EA group performed more platform crossings than mice in the AD group (*P* < 0.01, Figure 1(d)). There was a significant difference between the EA group and the NA group (*P* < 0.05).

**Figure 2:**
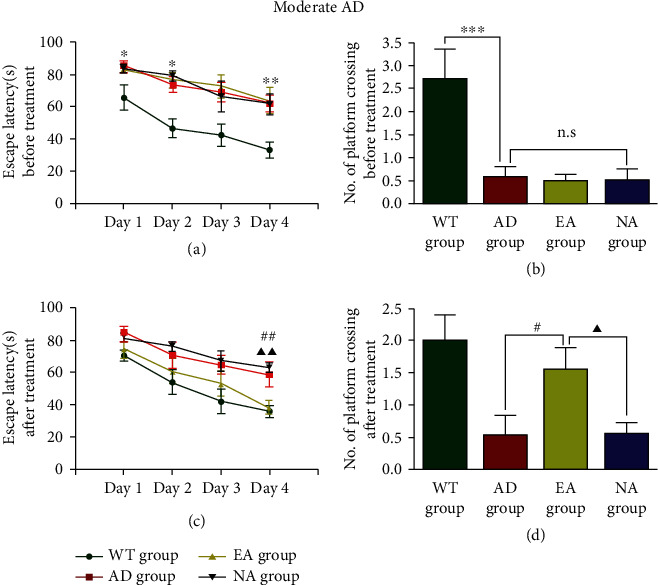
The Morris water maze test in moderate AD mice. Escape latency (a) and the number of platform crossings (b) before treatment. Escape latency (c) and the number of platform crossings (d) after treatment. *N* = 8/group, WT group vs AD group, ^∗^*P* < 0.05, ^∗∗^*P* < 0.01, ^∗∗∗^*P* < 0.001. AD group vs EA group, #*P* < 0.05. EA group vs NA group, ▲*P* < 0.05, ▲▲*P* < 0.01, n.s. means no significant difference.

In moderate AD, we also found that the WT group mice had a lower escape latency (*P* < 0.01, Figure 2(a)) and more platform crossing times (*P* < 0.001, Figure 2(b)) than the AD group. There was no significant difference between the AD group, the EA group, and the NA group at baseline. After treatment, mice in the EA group spent less time finding the platform than those in the AD group (*P* < 0.01, Figure 2(c)), and there was a significant difference between the EA group and the NA group (*P* < 0.01). Mice in the EA group showed more platform crossing times than mice in the AD group (*P* < 0.05, Figure 2(d)). There was a significant difference between the EA group and the NA group (*P* < 0.05).

These data indicated that APP/PS1 mice showed learning and memory deficits at an early age that worsened with increasing age. EA at DU24 and DU20 delayed learning and memory impairment in mice with mild and moderate AD.

### 3.2. Electroacupuncture Reduced A*β* Plaques in APP/PS1 Transgenic Mice

**Figure 3:**
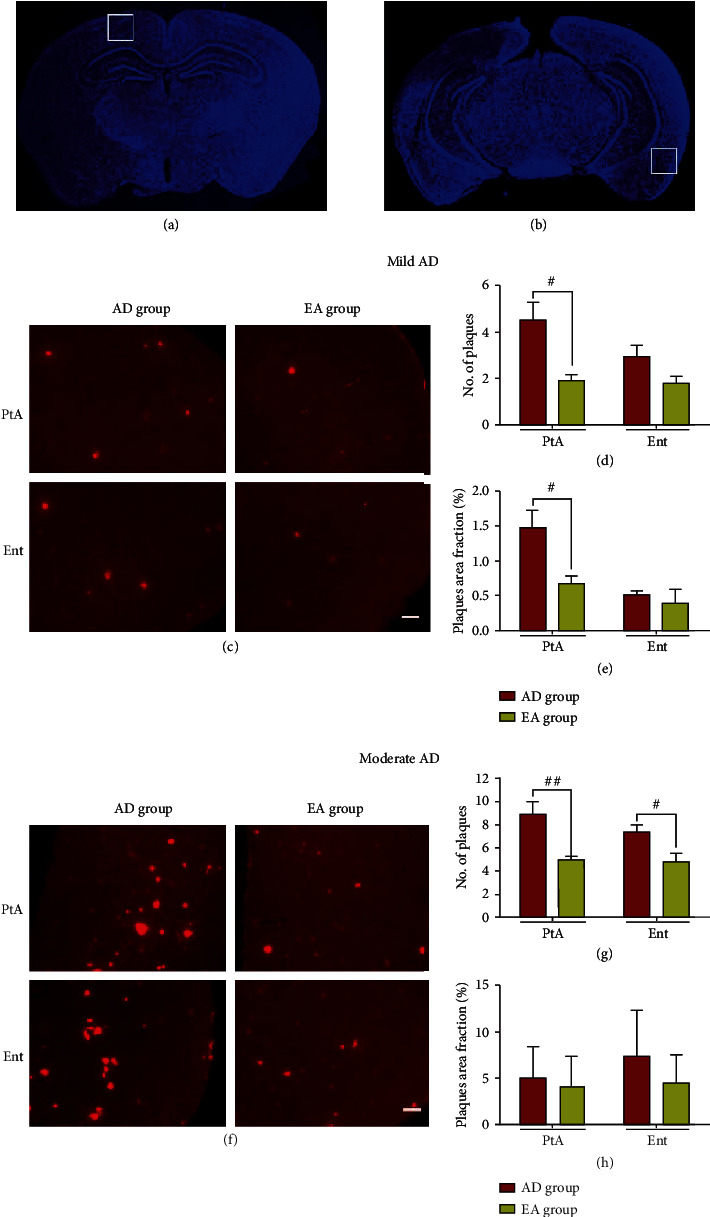
Electroacupuncture reduced A*β* plaques. The localization map of PtA (a) and Ent (b). Immunofluorescence with anti-A*β* (6E10) antibody in the PtA and Ent of different groups in mild AD mice (c). The average number of A*β*-positive plaques (d) and average A*β*-positive plaques area fraction (e) of each field of view. A*β*-positive plaques in the PtA and Ent of different groups in moderate AD mice (f). The average number of A*β*-positive plaques (g) and average A*β*-positive plaques area fraction (h) of each field of view. Scale bar = 100 *μ*m, *n* = 4/group, #*P* < 0.05, ##*P* < 0.01.

Given that the EA stimulation could improve learning and memory in APP/PS1 mice, we next assessed its effects on A*β* plaques and performed immunofluorescence with 6E10. The localization map of PtA and Ent was shown as 3a and 3b. For mild AD mice, the representative pictures of A*β*-positive plaques were shown in Figure 3(c). APP/PS1 mice with EA showed a lower number and area fraction of A*β*-positive plaques in the PtA than AD mice (*P* < 0.05, Figures 3(d) and 3(e)). There was no significant difference in A*β* plaque number or area fraction in the Ent between the AD group and the EA group. For moderate AD mice, the representative pictures of A*β*-positive plaques were shown in Figure 3(f). We found that the EA group showed a reduced A*β*-positive plaque number in the PtA and Ent compared with the AD group (*P* < 0.01 and *P* < 0.05, respectively, Figure 3(g)). However, there was no significant difference in the plaque area fraction between the AD group and EA group in the PtA and Ent (Figure 3(h)).

Collectively, these results demonstrated that EA at DU24 and DU20 could reduce the amyloid load in PtA of mild AD mice.

### 3.3. Electroacupuncture Transformed the Microglial Polarization State in APP/PS1 Transgenic Mice

**Figure 4:**
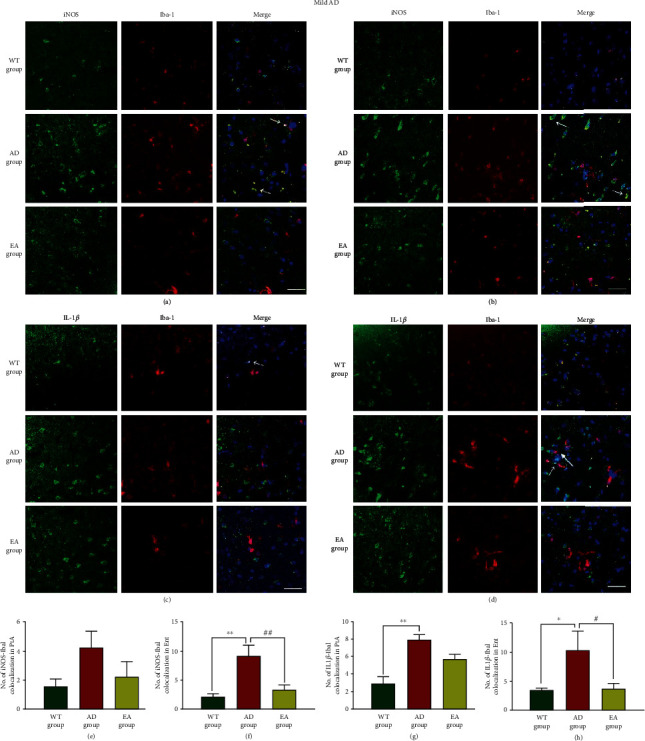
The colocalization of M1 markers and microglia in mild AD mice. Representative images of iNOS-Iba1 (a, b) and IL-1*β*-Iba1 (c, d) are shown in different areas. The immunofluorescence expression of the iNOS-Iba1 colocalization in the PtA (a) and Ent (b) and the number of colocalization in the PtA (e) and Ent (f). The immunofluorescence expression of the IL-1*β*-Iba1 colocalization in the PtA (c) and Ent (d) and the number of colocalization in the PtA (g) and Ent (h). Scale bar = 50 *μ*m, *n* = 4/group, WT group vs AD group, ^∗^*P* < 0.05, ^∗∗^*P* < 0.01. AD group vs EA group, #*P* < 0.05, ##*P* < 0.01.

**Figure 5:**
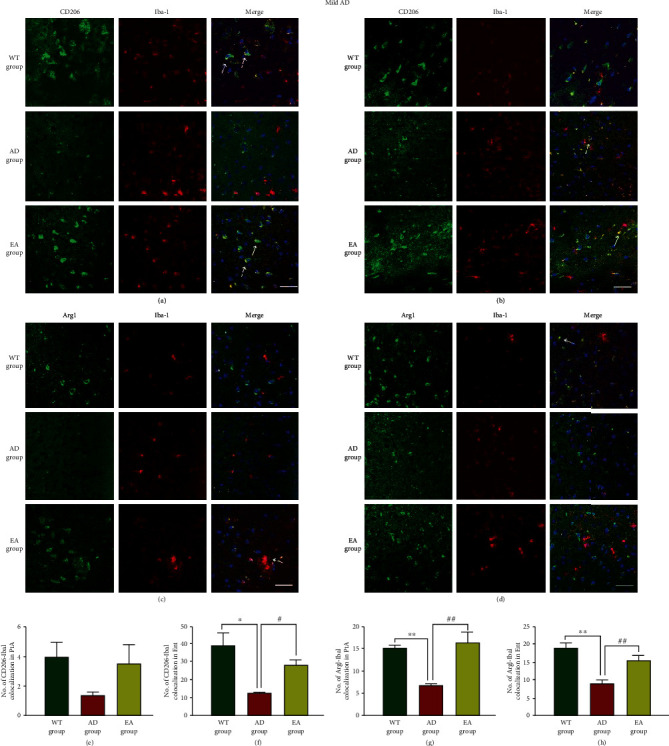
The colocalization of M2 markers and microglia in mild AD mice. Representative images of CD206-Iba1 (ab) and Arg1-Iba1 (cd) are shown in different areas. The immunofluorescence expression of the CD206-Iba1 colocalization in the PtA (a) and Ent (b) and the number of colocalization in the PtA (e) and Ent (f). The immunofluorescence expression of the Arg1-Iba1 colocalization in the PtA (c) and Ent (d) and the number of colocalization in the PtA (g) and Ent (h). Scale bar = 50 *μ*m, *n* = 4/group, WT group vs AD group, ^∗^*P* < 0.05, ^∗∗^*P* < 0.01. AD group vs EA group, #*P* < 0.05, ##*P* < 0.01.

Having shown that the EA stimulation reduced A*β* plaques, we next aimed to investigate whether the EA stimulation could regulate the microglial polarization state and induce the expression of different phenotypes. Microglia were labeled with Iba-1, and we examined the morphological features of microglia (M1 microglia produce iNOS/IL-1*β*, and M2 microglia express CD206/Arg1) in response to the EA intervention. In mild AD, the representative pictures of the iNOS and IL-1*β* colocalization with microglia in the PtA and Ent were shown in Figures 4(a)–4(d). We observed that iNOS-Iba1 (*P* < 0.01 in the Ent, Figure 4(f)) and IL-1*β*-Iba1 (*P* < 0.01 in the PtA, *P* < 0.05 in the Ent, Figures 4(g) and 4(h)) colocalizations in AD mice were significantly increased compared with those in their age-matched WT counterparts. The EA group showed a 63.96% and 64.22% decline in the iNOS-Iba1 and IL-1*β*-Iba1 colocalizations, respectively, in the Ent (*P* < 0.01, Figure 4(f) and *p* < 0.05, Figure 4(h), respectively). The representative pictures of the CD206 and Arg1 colocalization with microglia in the PtA and Ent were shown in Figures 5(a)–5(d). In the AD group mice, the CD206-Iba1 colocalization (*P* < 0.05 in the Ent, Figure 5(f)) and Arg1-Iba1 colocalization (*P* < 0.01 in the PtA and Ent, Figures 5(g) and 5(h)) were significantly reduced compared with those in the WT group. The CD206-Iba1 colocalization increased 55.56% in the Ent (*P* < 0.05, Figure 5(f)), and the Arg1-Iba1 colocalization increased 55.55% and 42.14% in the PtA and Ent, respectively (*P* < 0.01, Figures 5(g) and 5(h)).

**Figure 6:**
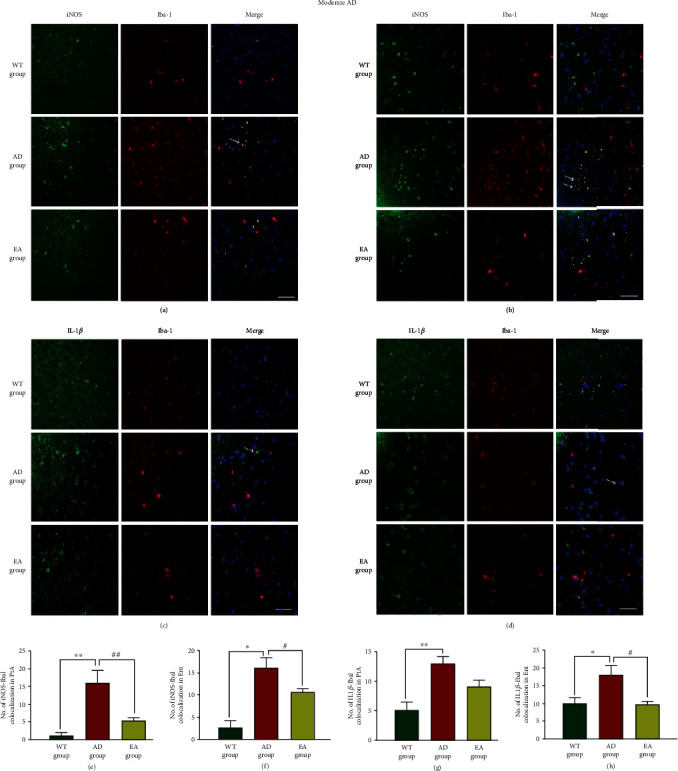
The colocalization of M1 markers and microglia in moderate AD mice. Representative images of iNOS-Iba1 (a, b) and IL-1*β*-Iba1 (c, d) are shown in different areas. The immunofluorescence expression of the iNOS-Iba1 colocalization in the PtA (a) and Ent (b) and the number of colocalization in the PtA (e) and Ent (f). The immunofluorescence expression of the IL-1*β*-Iba1 colocalization in the PtA (c) and Ent (d) and the number of colocalization in the PtA (g) and Ent (h). Scale bar = 50 *μ*m, *n* = 4/group, WT group vs AD group, ^∗^*P* < 0.05, ^∗∗^*P* < 0.01. AD group vs EA group, #*P* < 0.05, ##*P* < 0.01.

**Figure 7:**
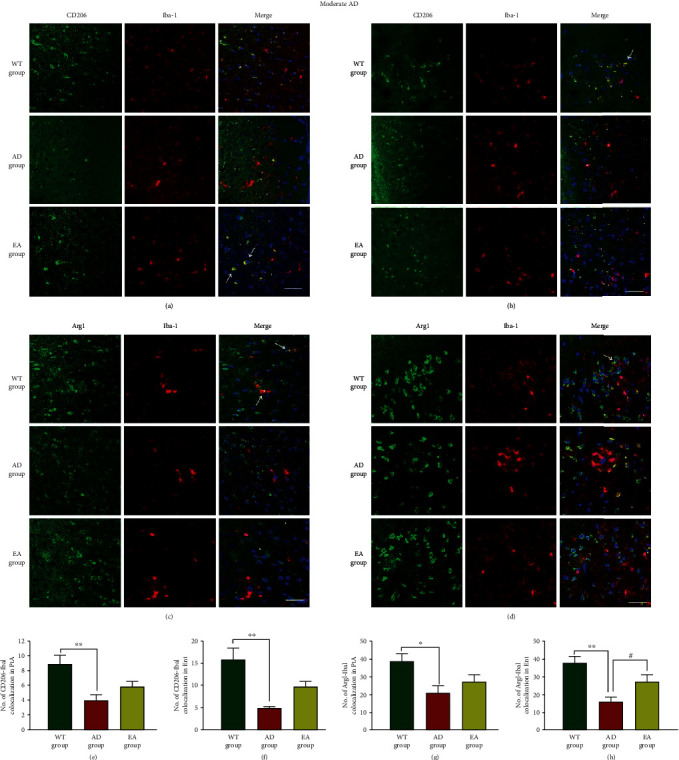
The colocalization of M2 markers and microglia in moderate AD mice. Representative images of CD206-Iba1 (a, b) and Arg1-Iba1 (c, d) are shown in different areas. The immunofluorescence expression of the CD206-Iba1 colocalization in the PtA (a) and Ent (b) and the number of colocalization in the PtA (e) and Ent (f). The immunofluorescence expression of the Arg1-Iba1 colocalization in the PtA (c) and Ent (d) and the number of colocalization in the PtA (g) and Ent (h). Scale bar = 50 *μ*m, *n* = 4/group, WT group vs AD group, ^∗^*P* < 0.05, ^∗∗^*P* < 0.01. AD group vs EA group, #*P* < 0.05.

In moderate AD, the representative pictures of the iNOS and IL-1*β* colocalization with microglia in the PtA and Ent were shown in Figures 6(a)–6(d). We found that the iNOS-Iba1 colocalization and IL-1*β*-Iba1 colocalization in AD mice were decreased compared with those in the WT group (*P* < 0.01 in the PtA, Figures 6(e) and 6(g) and *p* < 0.05 in the Ent, Figures 6(f) and 6(h)). EA at DU24 and DU20 reduced the iNOS-Iba1 colocalization in all areas (55.36% in the PtA, *P* < 0.01, Figure 6(e); 32.94% in the Ent, *P* < 0.05, Figure 6(f)) and the IL-*β*-Iba1 colocalization in the Ent (45.62%, *P* < 0.05, Figure 6(h)) compared with AD mice. The representative pictures of the CD206 and Arg1 colocalization with microglia in the PtA and Ent are shown in Figures 7(a)–7(d). The CD206-Iba1 coexpression and Arg1-Iba1 coexpression were decreased in the PtA and Ent in the AD group compared with the WT group (*P* < 0.01 or *P* < 0.05, Figures 7(e)–7(h)). There was no significant difference in CD206-Iba1 in the PtA and Ent between the EA group and the AD group (Figures 7(e) and 7(f)). EA at DU20 and DU24 increased the Arg1-Iba1 colocalization in the Ent compared with the AD group (*P* < 0.01, Figure 7(h)).

These results demonstrated that EA at DU24 and DU20 could regulate the microglial polarization state, promoting microglia to the M1 phenotype and inhibiting microglia to the M2 phenotype. Moreover, these effects were more evident in mild AD mice.

### 3.4. Electroacupuncture Regulated M1/M2 mRNA Levels in APP/PS1 Transgenic Mice

**Figure 8:**
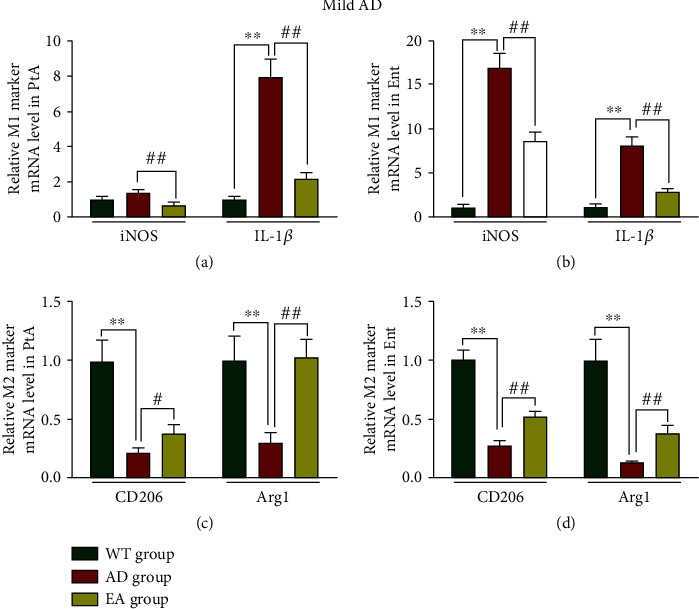
Relative M1 and M2 marker mRNA levels in mild AD mice. M1 marker mRNA levels in the PtA (a) and Ent (b). M2 marker mRNA levels in the PtA (c) and Ent (d). *N* = 4/group, WT group vs AD group, ^∗∗^*P* < 0.01. AD group vs EA group, #*P* < 0.05, ##*P* < 0.01.

We measured the M1 (iNOS/IL-1*β*) and M2 (CD206/Arg1) mRNA levels by qPCR. In mild AD, we found that IL-1*β* mRNA levels were increased (*5*, Figures 8(a) and 8(b)), and CD206 and Arg1 mRNA levels were reduced (*P* < 0.01, Figures 8(c) and 8(d)) in the AD group compared with the WT group. After the intervention, the mRNA levels of iNOS and IL-1*β* were reduced in the PtA and Ent (*P* < 0.01), and the mRNA levels of CD206 and Arg1 were increased in the PtA and Ent (*P* < 0.05 or *P* < 0.01) of the EA group.

**Figure 9:**
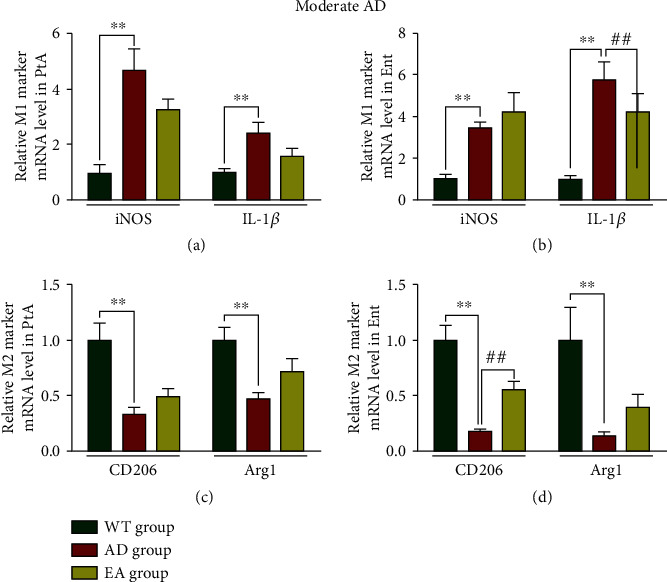
Relative M1 and M2 marker mRNA levels in moderate AD mice. M1 marker mRNA levels in the PtA (a) and Ent (b). M2 marker mRNA levels in the PtA (c) and Ent (d). *N* = 4/group, WT group vs AD group, ^∗∗^*P* < 0.01. AD group vs EA group, ##*P* < 0.01.

In moderate AD, iNOS and IL-1*β* mRNA levels were increased while CD206 and Arg1 mRNA levels were reduced in both the PtA and Ent in the AD group compared with the WT group (*P* < 0.01, Figures 9(a)–9(d)). There was no significant difference in iNOS, IL-1*β*, CD206, and Arg1 mRNA levels in the PtA between the AD group and the EA group. In the EA group, IL-1*β* mRNA levels were downregulated, and CD206 mRNA levels were upregulated in the Ent (*P* < 0.01) compared with the AD group.

Overall, our results showed that EA at DU24 and DU20 could regulate M1 and M2 marker mRNA levels in mild AD mice.

## 4. Discussion

In this study, we demonstrated that EA at DU24 and DU20 could improve learning and memory, drive the attenuation of A*β*, and regulate the microglial M1/M2 polarization in mild AD and moderate AD.

A progressive loss of memory and cognitive impairment is principal clinical presentations of AD, and the MWM test is a traditional methodology to measure learning and memory in animals. Research has suggested that a gradual decline in cognition could be detected in most 6- to 18-month-old APP/PS1 mice, and young (less than or equal to 5 months old) APP/SP1 mice showed little difference in the declining trend of escape latency after repeated training when compared with WT mice [32]. A study indicated that there was no significant difference in escape latency between WT and APP/PS1 mice at 3 months of age, while the escape latency was significantly longer in 5-month-old APP/PS1 mice than in their counterparts in the MWM test [33]. Therefore, we chose the 4-month-old APP/PS1 mice to simulate the mild AD stage. On the other hand, A*β* plaques were observed in APP/PS1 mice as young as 3 months of age, with linear increases with months of age, but slow growth was observed after 12 months of age [33]. Therefore, we chose the 12-month-old APP/PS1 mice to simulate the moderate AD stage.

DU24 and DU20 are located along the governor vessel, and their stimulation is widely reported to improve cognition [34–37]. Our previous studies proved that EA at DU24 and DU20 improved cognitive deficits [34] and mediated synaptic plasticity in the periinfarct hippocampal CA1 region of rats following ischaemic stroke [38]. Furthermore, the EA stimulation of DU24 and DU20 might reactivate cognition-related brain regions [37]. The Ent is the critical structure relaying memory-related information between the neocortex and the hippocampus, which is first affected in AD [39, 40]. The PtA is often considered to be the brain region with obvious pathological changes in neuroimaging [41]. Evidence from [18F]flortaucipir PET and T1-weighted magnetic resonance imaging showed that the lateral and medial PtA and lateral temporal cortex were most relevant to cognitive decline in AD [42].

A*β*, reactive gliosis, and neuroinflammation are hallmarks of AD [43]. Long considered to be secondary events to neurodegeneration, microglia-related pathways have been identified as central to AD risk and pathogenesis [44, 45]. We proved the bidirectional regulation of the DU24 and DU20 stimulation on the microglial polarization: it downregulated the M1 phenotype microglia and upregulated the M2 phenotype microglia in mild AD. EA inhibited iNOS and IL-1*β* and promoted CD206 and Arg1 in 8 months old APP/PS1 mice. However, EA could not upregulate the M2 phenotype microglia in moderate AD. We found that the iNOS and IL-1*β* colocalization decreased, but CD206 and Arg1 had no difference in immunofluorescence and mRNA level in 16 months old APP/PS1 mice. It could expound that the effect of EA on AD mice at different stages is different. Therefore, EA was more effective in mild AD mice than in moderate AD mice. EA suppressed not only the microglial polarization to the M1 phenotype but also promoted the microglial polarization to the M2 phenotype in mild AD mice.

Microglia can be neuroprotective by degrading A*β* plaques similar to action against the A*β* accumulation [46]. However, an age-dependent increase in both the number and the size of A*β* plaques in AD might reflect a diminution in the microglial phagocytic capability [47]. In the present study, we found that EA at DU24 and DU20 reduced A*β*-positive plaques in the PtA, rather than Ent in mild AD mice. It may be related to the targeting brain region under different acupoints. Research had proved that EA at DU20 and right Qubin (GB7, acupuncture) could increase glucose metabolism in the parietal lobe [48]. However, we found changes only in the number of A*β*-positive plaques, not in the area fraction in moderate AD mice. It may indicate that the effect of EA is limited, which could not eliminate the accumulation of A*β* in 16 months old APP/PS1 mice. Moreover, it needs further study.

## 5. Conclusion

EA at DU24 and DU20 reduced A*β* plaques to improve learning and memory in mild AD mice. This finding is mainly related to the microglial M1/M2 polarization inducing by EA.

## Data Availability

The data used to support the findings of this study are available from the corresponding author upon request.

## References

[1] Tramutola A., Lanzillotta C., Perluigi M., Butterfield D. A. (2017). Oxidative stress, protein modification and Alzheimer disease. *Brain Research Bulletin*.

[2] Prince M. (2015). *World Alzheimer Report: The Global Impact of Dementia*.

[3] Schneider L. S., Mangialasche F., Andreasen N. (2014). Clinical trials and late-stage drug development for Alzheimer’s disease: an appraisal from 1984 to 2014. *Journal of Internal Medicine*.

[4] Ott B. R. (2013). The long and winding road toward Alzheimer prevention FDA offers new guidance on developing drugs for early-stage AD; seeks input. *R.I. Medical Journal*.

[5] Schneider L. S. (2014). Rethinking the Food and Drug Administration's 2013 guidance on developing drugs for early-stage Alzheimer's disease. *Alzheimers Dement*.

[6] Kawakita K., Okada K. (2014). Acupuncture therapy: mechanism of action, efficacy, and safety: a potential intervention for psychogenic disorders. *Biopsychosoc Med*.

[7] Lu W., Dean-Clower E., Doherty-Gilman A., Rosenthal D. S. (2008). The value of acupuncture in cancer care. *Hematology/Oncology Clinics of North America*.

[8] Jia Y., Zhang X., Yu J. (2017). Acupuncture for patients with mild to moderate Alzheimer's disease: a randomized controlled trial. *BMC Complementary and Alternative Medicine*.

[9] Shao S., Tang Y., Guo Y., Tian Z., Xiang D., Wu J. (2019). Effects of acupuncture on patients with Alzheimer's disease: protocol for a systematic review and meta-analysis. *Medicine (Baltimore)*.

[10] Feng Q., Bin L. L., Zhai Y. B., Xu M., Liu Z. S., Peng W. N. (2019). Long-term efficacy and safety of electroacupuncture on improving MMSE in patients with Alzheimer's disease. *Zhongguo Zhen Jiu*.

[11] Dong W., Guo W., Zheng X. (2015). Electroacupuncture improves cognitive deficits associated with AMPK activation in SAMP8 mice. *Metabolic Brain Disease*.

[12] Zhang M., Xv G. H., Wang W. X., Meng D. J., Ji Y. (2017). Electroacupuncture improves cognitive deficits and activates PPAR-*γ* in a rat model of Alzheimer's disease. *Acupuncture in Medicine*.

[13] Zhao J., Wang L., Li Y. (2017). Electroacupuncture alleviates the inflammatory response via effects on M1 and M2 macrophages after spinal cord injury. *Acupuncture in Medicine*.

[14] Deng J., Lv E., Yang J. (2015). Electroacupuncture remediates glial dysfunction and ameliorates neurodegeneration in the astrocytic *α*-synuclein mutant mouse model. *Journal of Neuroinflammation*.

[15] Parkhurst C. . N., Yang G., Ninan I. (2013). Microglia promote learning-dependent synapse formation through brain-derived neurotrophic factor. *Cell*.

[16] Udeochu J. C., Shea J. M., Villeda S. A. (2016). Microglia communication: parallels between aging and Alzheimer's disease. *Clin Exp Neuroimmunol*.

[17] Czeh M., Gressens P., Kaindl A. M. (2011). The yin and yang of microglia. *Developmental Neuroscience*.

[18] Prinz M., Priller J., Sisodia S. S., Ransohoff R. M. (2011). Heterogeneity of CNS myeloid cells and their roles in neurodegeneration. *Nature Neuroence*.

[19] Jimenez S., Baglietto-Vargas D., Caballero C. (2008). Inflammatory response in the hippocampus of PS1M146L/APP751SL mouse model of Alzheimer's disease: age-dependent switch in the microglial phenotype from alternative to classic. *The Journal of Neuroscience*.

[20] Nathan C., Calingasan N., Nezezon J. (2005). Protection from Alzheimer's-like disease in the mouse by genetic ablation of inducible nitric oxide synthase. *The Journal of Experimental Medicine*.

[21] Chhor V., Le Charpentier T., Lebon S. (2013). Characterization of phenotype markers and neuronotoxic potential of polarised primary microglia in vitro. *Brain Behavior & Immunity*.

[22] Hu X., Leak R. K., Shi Y. (2015). Microglial and macrophage polarization—new prospects for brain repair. *Nature Reviews. Neurology*.

[23] Yang Z., Kuboyama T., Tohda C. (2019). Naringenin promotes microglialM2polarization andA*β*degradation enzyme expression. *Phytotherapy Research*.

[24] Iwahara N., Hisahara S., Kawamata J. (2017). Role of suppressor of cytokine signaling 3 (SOCS3) in altering activated microglia phenotype in APPswe/PS1dE9 mice. *Journal of Alzheimer's Disease*.

[25] Liu W., Zhuo P., Li L. (2017). Activation of brain glucose metabolism ameliorating cognitive impairment in APP/PS1 transgenic mice by electroacupuncture. *Free Radical Biology & Medicine*.

[26] Lin R., Li L., Zhang Y. (2018). Electroacupuncture ameliorate learning and memory by improving N-acetylaspartate and glutamate metabolism in APP/PS1 mice. *Biological Research*.

[27] Han B., Lu Y., Zhao H., Wang Y., Wang T. (2015). Electroacupuncture modulated the inflammatory reaction in MCAO rats via inhibiting the TLR4/NF-*κ*B signaling pathway in microglia. *International Journal of Clinical & Experimental Pathology*.

[28] Jiang J., Luo Y., Qin W. (2017). Electroacupuncture suppresses the NF-*κ*B signaling pathway by upregulating cylindromatosis to alleviate inflammatory injury in cerebral ischemia/reperfusion rats. *Frontiers in Molecular Neuroscience*.

[29] Condello C., Yuan P., Grutzendler J. (2018). Microglia-mediated neuroprotection, TREM2 , and Alzheimer's disease: evidence from Optical Imaging. *Biological Psychiatry*.

[30] Huang S., Lil L., Wang Z. (2019). Effect of electroacupuncture on learning-memory and expression of ß-site amyloid precursor protein cleaving enzyme 1 in APP/PSl mice. *Chin J Rehabil Theory Pract*.

[31] George P., BJF K. (2001). *The mouse brain in Stereotaxic Coordinates (second edition)*.

[32] Gao T. (2015). A review on the relationship between AP level in brain and age-related changes of cognitive behavior in APP / PS1 mice. *JOURNAL OF APOPLEXY AND NERVOUS DISEASES*.

[33] Zhu S., Wang J., Zhang Y. (2017). The role of neuroinflammation and amyloid in cognitive impairment in an APP/PS1 transgenic mouse model of Alzheimer's disease. *CNS Neuroscience & Therapeutics*.

[34] Lin R., Yu K., Li X. (2016). Electroacupuncture ameliorates post-stroke learning and memory through minimizing ultrastructural brain damage and inhibiting the expression of MMP-2 and MMP-9 in cerebral ischemia-reperfusion injured rats. *Molecular Medicine Reports*.

[35] Ye Y., Li H., Yang J. W. (2017). Acupuncture attenuated vascular dementia-induced hippocampal long-term potentiation impairments via activation of D1/D5 receptors. *Stroke*.

[36] Zhang Q., Li Y. N., Guo Y. Y. (2017). Effects of preconditioning of electro-acupuncture on postoperative cognitive dysfunction in elderly: a prospective, randomized, controlled trial. *Medicine (Baltimore)*.

[37] Wen T., Zhang X., Liang S. (2018). Electroacupuncture ameliorates cognitive impairment and spontaneous low-frequency brain activity in rats with ischemic stroke. *Journal of Stroke and Cerebrovascular Diseases*.

[38] Xie G., Song C., Lin X. (2019). Electroacupuncture regulates hippocampal synaptic plasticity via inhibiting Janus-activated kinase 2/signal transducer and activator of transcription 3 signaling in cerebral ischemic rats. *Journal of Stroke and Cerebrovascular Diseases*.

[39] Nakazono T., Lam T. N., Patel A. Y. (2017). Impaired In Vivo Gamma Oscillations in the Medial Entorhinal Cortex of Knock-in Alzheimer Model. *Frontiers in Systems Neuroscience*.

[40] Li X.-Y., Men W.-W., Zhu H. (2016). Age- and Brain Region-Specific Changes of Glucose Metabolic Disorder, Learning, and Memory Dysfunction in Early Alzheimer’s Disease Assessed in APP/PS1 Transgenic Mice Using 18F-FDG-PET. *International Journal of Molecular Ences*.

[41] Mase M., Nagai H., Kabasawa H., Ogawa T., Iida A., Yamada K. (2004). Cerebral blood flow and metabolism in patients with cognitive impairments after minor traumatic brain injury: PET study in a chronic state. *International Congress*.

[42] Ossenkoppele R., Smith R., Ohlsson T. (2019). Associations between tau, A*β*, and cortical thickness with cognition in Alzheimer disease. *Neurology*.

[43] Wyss-Coray T., Rogers J. (2012). Inflammation in Alzheimer disease-a brief review of the basic science and clinical literature. *Cold Spring Harbor Perspectives in Medicine*.

[44] Jonsson T., Stefansson H., Steinberg S. (2013). Variant of TREM2 associated with the risk of Alzheimer's disease. *New England Journal of Medicine*.

[45] Zhang B., Gaiteri C., Bodea L.-G. (2013). Integrated systems approach identifies genetic nodes and networks in late-onset Alzheimer's disease. *Cell*.

[46] Takata K., Kitamura Y., Saeki M. (2010). Galantamine-induced amyloid-beta clearance mediated via stimulation of microglial nicotinic acetylcholine receptors. *The Journal of Biological Chemistry*.

[47] Mawuenyega K. G., Sigurdson W., Ovod V. (2010). Decreased clearance of CNS beta-amyloid in Alzheimer's disease. *Science*.

[48] Fang Z., Ning J., Xiong C., Shulin Y. (2012). Effects of electroacupuncture at head points on the function of cerebral motor areas in stroke patients: a PET study. *Evidence-based Complementary and Alternative Medicine*.

